# BioVizSeq: an R package for visualization the element on bio-sequences

**DOI:** 10.3389/fpls.2025.1645004

**Published:** 2025-08-19

**Authors:** Shiqi Zhao, Runqi Zhang, Maoqiu He, Bonian Shui, Yu Zhang

**Affiliations:** School of Fishery, Zhejiang Ocean University, Zhoushan, China

**Keywords:** BioVizSeq, biosequence, element, visualization, shinyApp

## Abstract

The identification and visualization of functional elements within biological sequences offers visual presentation for biologists to integrate annotation, and also helps them to produce high-quality figures for publication. Although there are now some standalone tools that can perform this function, these tools generally lack flexibility and cannot meet personalized needs. Based on the advantages of R language in graphic display, we have developed an R package: BioVizSeq (CRAN: https://cran.r-project.org/package=BioVizSeq. Github: https://github.com/zhaosq2022/BioVizSeq). It is designed for visualizing the types and distribution of elements within bio-sequences. These data could come from users or analysis programs, such as, GFF/GTF, MEME, SMART, Plantcare, PFAM, CDD and etc. BioVizSeq can be conducted locally or online, providing great convenience for researchers without coding training. Its user-friendly visualization function can simultaneously meet users’ general needs and personalized exploration.

## 1 Introduction

Biological sequences typically refer to nucleotide sequences (DNA or RNA) or protein sequences, which encode the genetic information of an organism. Nucleotide sequences contain coding regions (open reading frames, ORFs), regulatory elements (such as promoters, enhancers, silencers), splice sites, transcription factor binding sites, and replication origins. Protein sequences, composed of amino acids, fold into specific three-dimensional structures or multi-subunit complexes to carry out various biological functions. Functional motifs or domains in proteins include signal peptides, enzymatic active sites, binding sites, phosphorylation sites, and transmembrane regions, all of which are critical for the protein’s function (Savojardo et al., 2023). In summary, the functional elements and motifs within biological sequences interact to facilitate a wide range of physiological processes in living organisms.

The identification and visualization of functional elements within biological sequences offers visual presentation for biologists to integrate annotation, and also helps them to produce high-quality figures for publication. For nucleic acid sequence structures, GTF (Gene Transfer Format) and GFF (General Feature Format) files are commonly used for display (Pertea and Pertea, 2020). PlantCare is typically used to analyze and predict cis-regulatory elements on plant gene promoter sequences, but there is no similar tool for animal gene promoters at present (Lescot et al., 2002). For functional elements on protein sequences, tools such as PFAM (Mistry et al., 2021), SMART (Letunic et al., 2021), NCBI-CDD (Wang et al., 2023), or MEME (Bailey et al., 2015) are commonly used for analysis and prediction. To better observe the similarities and differences of these elements across different subfamilies and explore gene evolution, these structures are often compared with evolutionary trees. In terms of tools for displaying and combining these structures, there are standalone tools like TBtools (Chen et al., 2020, 2023) and CFVisual (Chen et al., 2022). However, the disadvantages of standalone software tools are also present in these tools, such as limited flexibility, low automation, and platform restrictions.

R language has numerous advantages in data analysis and visualization. In terms of data analysis, it offers a wide range of third-party packages for tasks such as data cleaning and transformation. Additionally, R excels in data visualization capabilities; through plotting packages like ggplot2 (Klaus and Galensa, 2017), it can generate professional and complex graphics with strong customization options. Furthermore, tools like the shiny package enable the creation of interactive graphics, allowing users to interact with the visualizations. Based on these features, we developed a biosequence element visualization package in R: BioVizSeq. In this package, we have written multiple functions for data analysis and visualization. Based on these functions, we have also developed a Shiny app within the package. Theoretically, BioVizSeq is not limited to the visualization of biological sequences. This package not only meets the interactive needs of general users but also supports the personalized display needs of advanced users.

## 2 Results and discussion

### 2.1 Overview of BioVizSeq

**Figure 1:**
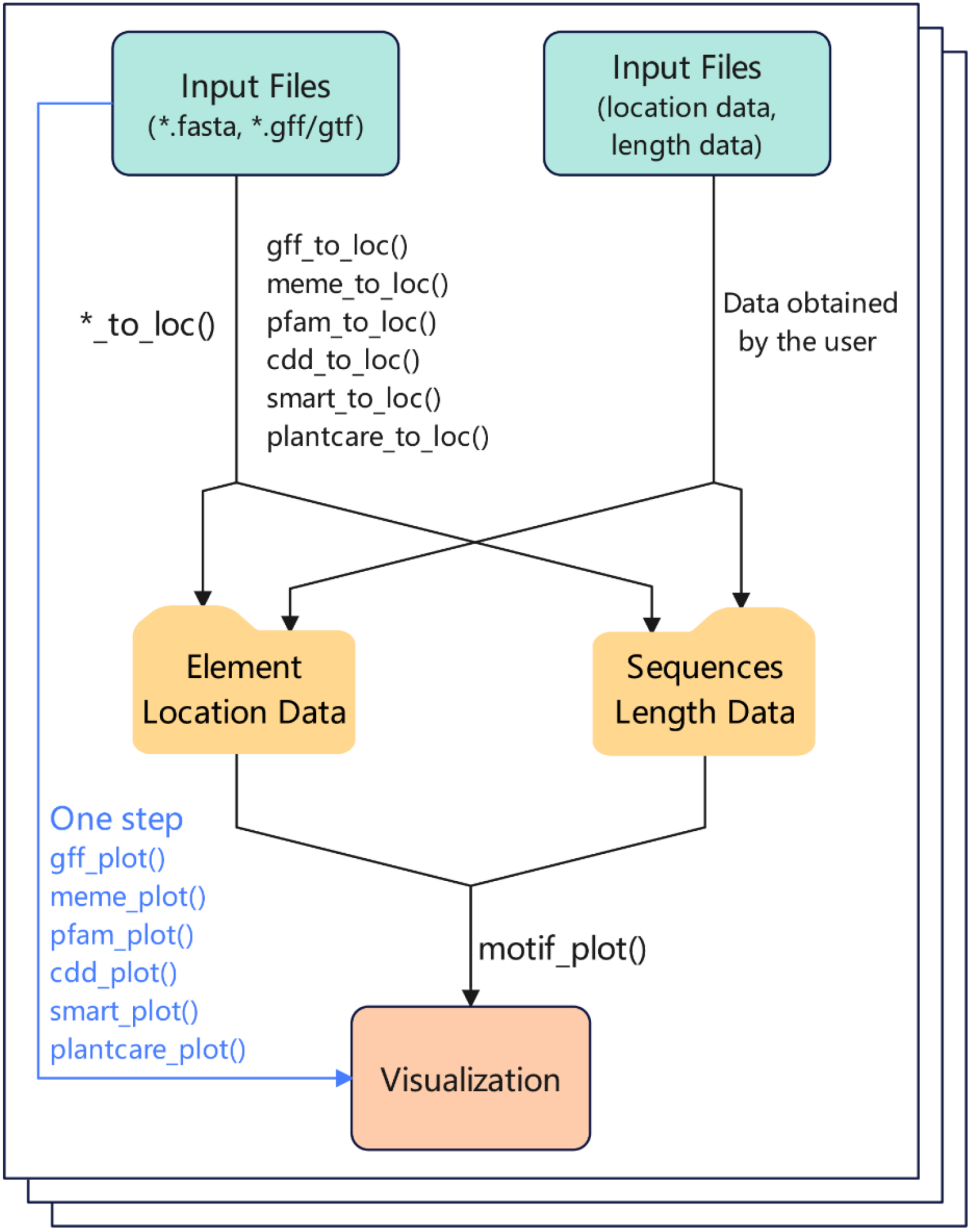
The basic workflow of BioVizSeq package. Input file: result files of software or databases (e.g.: MEME, SMART, PFAM, etc.), or manually organized location file.

The BioVizSeq package is designed for visualizing the types and distribution of elements within bio-sequences. These data could come from users or analysis programs, such as, GFF/GTF, MEME, SMART, Plantcare, Pfam, CDD and etc (**Figure 1**). The BioVizSeq provides two modes for displaying: “Step by Step” and “One Step”. Since ggplot2 does not provide a geom for generating rounded rectangles, we have written a geom layer that can draw rounded rectangles: geom_rrect. There is a special function BioVizSeq to start our shinyapp interface system, specially customized for the BioVizSeq R package.

### 2.2 BioVizSeq resources

BioVizSeq is open source under Artistic-2.0 license, and all source codes of R package, API documentation, websites, and Shinyapp are stored in the GitHub repository https://github.com/zhaosq2022/BioVizSeq. BioVizSeq provides a stable version for multi‐platform installation on the Comprehensive R Archive Network (CRAN) website (https://cran.r-project.org/package=BioVizSeq), and the latest unstable version is available on the GitHub repository. Furthermore, we have created comprehensive API documentation and tutorials, including text content (https://zhaosq2022.github.io/BioVizSeq/).

To elucidate the benefits of BioVizSeq, we compared its functionality and resources with several other commonly used software (**Table 1**). Standalone visualization tools typically offer predefined graphical elements, whereas R enables programmatic customization through its scripting interface. This difference may influence their applicability in scenarios requiring highly tailored visual outputs. TBtools II and CFVisual are desktop applications compiled in Java and Python, respectively. Both have natural advantages in operating desktop software, but there are also some drawbacks or shortcomings, such as requiring local installation, opaque data processing, and inability to personalize display. In contrast, BioVizSeq effectively addresses these issues. In addition, TBtools II also has a similar Basic plot feature, but CFVisual does not. Meanwhile, neither of these supports SMART result display.

**Table 1:** Performance comparison of benchmarked tools.

Comment Points	TBtools-II	CFVisual	BioVizSeq
Cross-platform (Linux, Mac OS, and Windows)	yes	no	yes
GUI (Graphical User Interface)	yes	yes	yes
Website	no	no	yes
Code writing	No need	No need	Optional
Programming language	Java	Python	R
Basic biosequence view	yes	no	yes
Gene Structure (gff3/gtf)	yes	yes	yes
MEME	yes	yes	yes
PFAM	yes	yes	yes
NCBI CDD	yes	yes	yes
SMART	no	no	yes
Plantcare	no	yes	yes
Plantcare advance plot	no	no	yes
Free combination	no	no	yes
Data visualization	no	no	yes
Long‐term maintenance	yes	no	yes
Link address (source code)	no	no	https://github.com/zhaosq2022/BioVizSeq/

### 2.3 BioVizSeq shinyApp practicality

**Figure 2:**
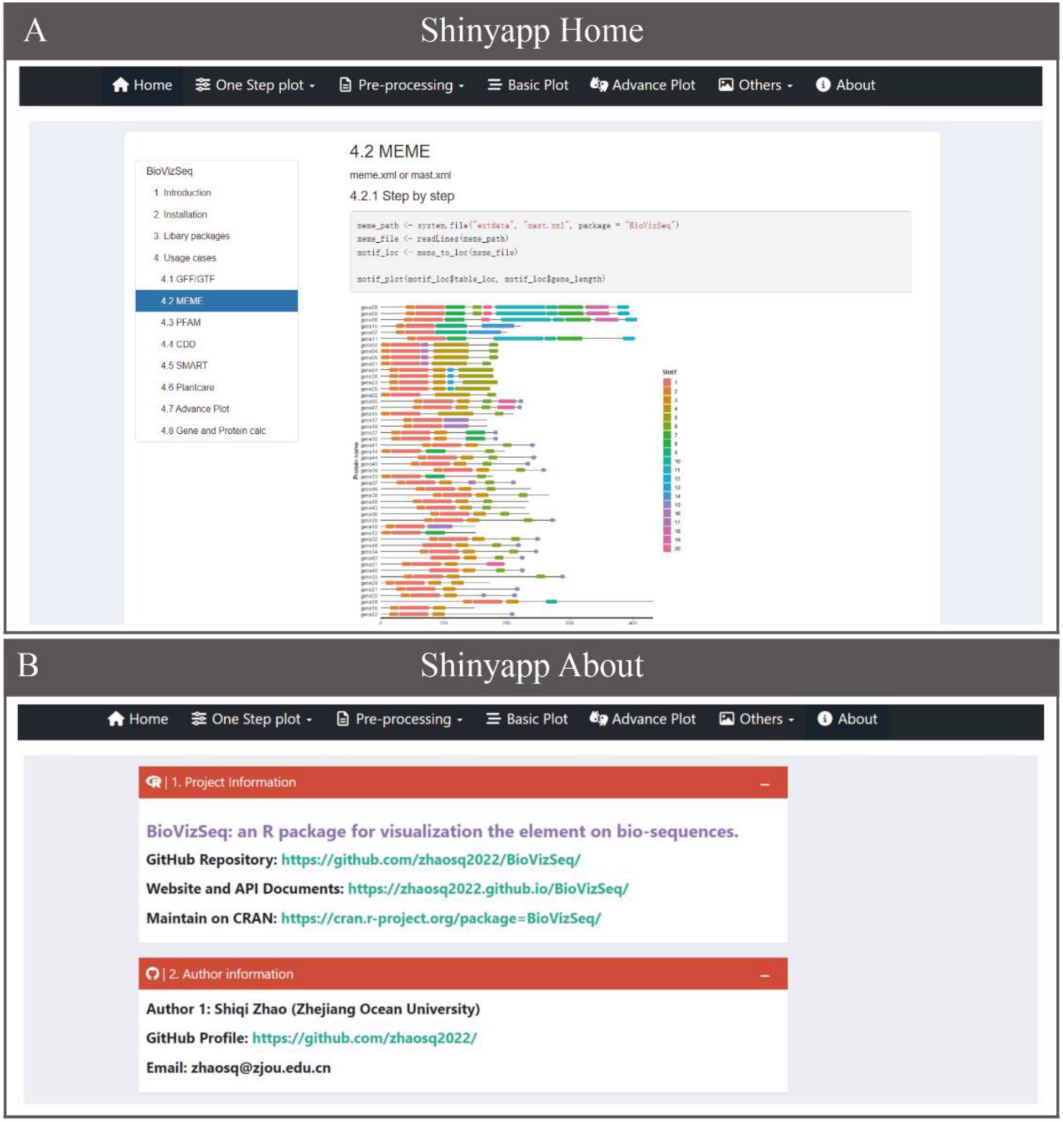
Overview of the shinyapp of BioVizSeq. **(A)** The Home. **(B)** The About.

To make BioVizSeq more accessible to researchers without coding experience, we have developed an intuitive interactive analysis and visualization platform based on Shiny v1.7.5 (https://github.com/rstudio/shiny/) and related packages (**Figure 2**). The BioVizSeq package allows users to install and run the tool locally, with all computations utilizing the local computer’s resources, including memory, CPU, and storage. Users can launch BioVizSeq’s shinyapp by executing the command ‘biovizsq()’. For added convenience, we also offer free online services and computational resources, enabling researchers to perform analysis tasks anytime and anywhere (https://myshiny.cpolar.io/BioVizSeq/ and https://mybase.vip.cpolar.cn/BioVizSeq/).

The BioVizSeq Shinyapp user interface includes the following sections: Home, One Step Plot, Pre-processing, Basic Plot, Advanced Plot, and About (**Figure 2**). The Home and About pages in the application menu display the BioVizSeq API documentation and project information, respectively. The other four collapsible menus contain applications that are organized by function categories, with each menu containing multiple modules. The parameter operation panel is divided into two main parts: the left side is used for data upload, analysis, visualization, and parameter download. The final result is displayed in a dedicated panel, where you can choose to download graphical and tabular results. In conclusion, the rapid deployment and stable performance of the BioVizSeq Shinyapp are supported by the out-of-the-box functionalities of BioVizSeq, which work together to facilitate long-term maintenance and further development.

### 2.4 User cases of BioVizSeq

#### 2.4.1 Basic plot

**Figure 3:**
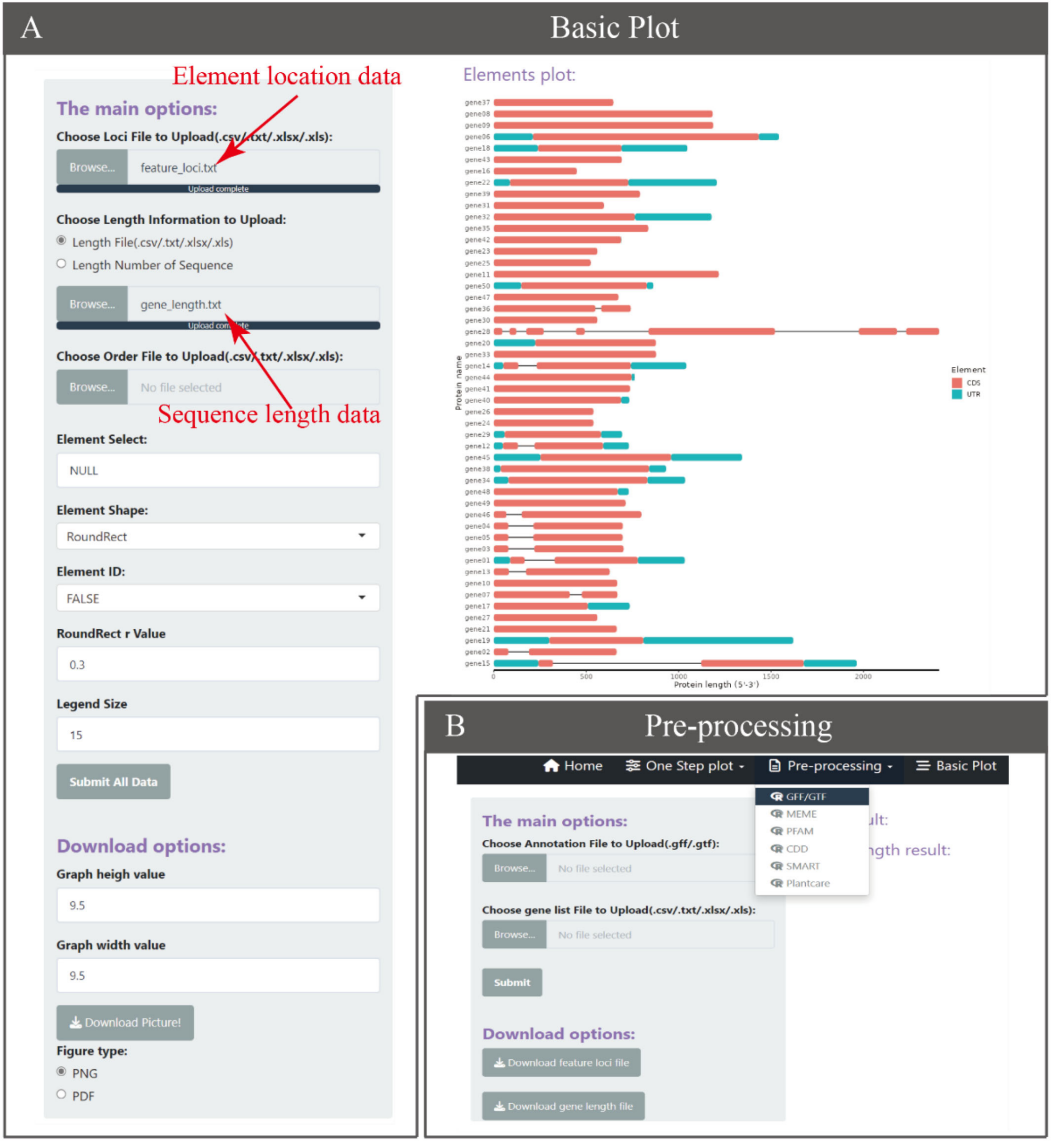
The Basic Plot function. **(A)** The Basic Plot operation and display interface. **(B)** Data preprocessing generates input data for the Basic Plot.

The core of BioVizSeq is to use the coordinate information of elements on the sequence and the length information of the sequence as input files, and use ggplot2 for graphic drawing. Based on this, we have developed a basic plot function motif_plot to facilitate users in graphic drawing (**Figure 3**). In theory, motif_plot can be graphically drawn using these two files for all sequences, whether they are proteins, DNA, or RNA sequences.

#### 2.4.2 Gene structure

**Figure 4:**
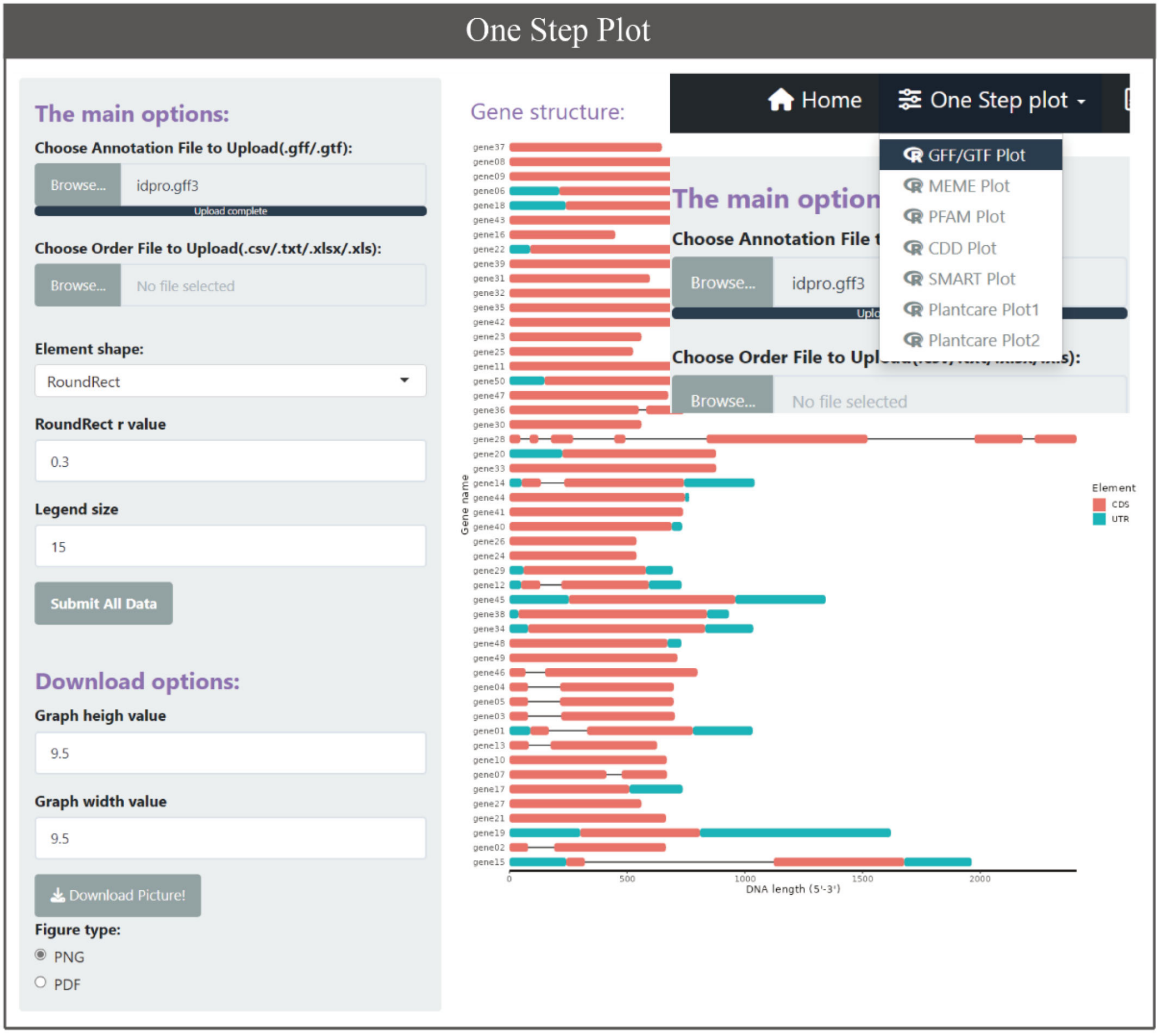
The one step plot function for GFF/GTF, MEME, PFAM, CDD, SMART, Plantcare.

GTF and GFF are popular file formats used by bioinformatics programs to represent and exchange information about various genomic features, such as gene and transcript locations and structure. There are currently multiple software or tools based on GFF3/GTF files for gene structure display, such as GSDS 2.0 (Hu et al., 2015), TBtools, etc. But there are no similar tools based on ggplot2 of R language yet. The BioVizSeq provides two modes for displaying gene structures through GFF3/GTF files. One approach is to divide the process into two steps. Firstly, the gene length information, UTR, and CDS information of the gene are obtained through gff_to_loc, and then the gene structure is freely drawn using motif_plot. Another approach is to directly use gff_plot to plot gene structure in one step, but with relatively limited freedom. (**Figure 4**).

#### 2.4.3 MEME motif

The MEME Suite web server (https://meme-suite.org/meme/tools/meme) is commonly used in bioinformatics to identify and display conserved patterns in DNA, RNA, or protein sequences. Its advantage lies in the ability to efficiently identify potential functional motifs and provide reliable significance evaluations for these motifs through statistical methods, helping researchers reveal important biological features in the sequence. However, MEME motifs also have certain drawbacks, especially in terms of display effectiveness. Firstly, the motif images generated by MEME cannot be downloaded. Secondly, the images generated by MEME tools are usually relatively single and difficult to flexibly combine or modify with other graphics or data, which limits their application in diversified graphic displays. In addition, the visual representation of motifs is relatively fixed and may not meet the expectations of aesthetic or customization needs in some specific studies. Fortunately, MEME Suite web server provides two result files: meme.xml and mast.xml. BioVizSeq first parses the result file, and then uses the motif plot function to plot the motif.

#### 2.4.4 PFAM domain

Pfam is a widely used protein family database that provides a wealth of information on protein domains and families. It is currently hosted by InterPro (https://www.ebi.ac.uk/interpro/), an integrated database that integrates resources from multiple protein sequences and domains (Blum et al., 2025). The “by sequence” feature in Search InterPro allows users to search for domains, and functional sites by submitting sequences. However, its display results are in the form of a table, which is not conducive to direct comparative observation. BioVizSeq can organize this result file and display the domain.

#### 2.4.5 CDD domain

NCBI’s Batch CD Search (https://www.ncbi.nlm.nih.gov/Structure/bwrpsb/bwrpsb.cgi) is a powerful tool that allows users to submit multiple protein sequences in batches and perform conservative domain searches and annotations on these sequences using the Conserved Domain Database (CDD). But its display results are also in tables. Therefore, BioVizSeq has developed the ability to showcase its results.

#### 2.4.6 SMART domain

SMART (a Simple Modular Architecture Research Tool) (https://smart.embl.de/) allows the identification and annotation of genetically mobile domains and the analysis of domain architectures. It adopts a modular architecture that can quickly and accurately identify structural domains in protein sequences, and has been widely used in bioinformatics research. However, SMART usually requires a certain foundation of Linux operating system when analyzing protein sequences in batches. The results returned in bulk are text files, which are very unfavorable for displaying the results. BioVizSeq can automatically upload sequences in bulk and organize and plot the returned results, greatly reducing the operational threshold.

#### 2.4.7 Plantcare *cis*-acting regulatory elements

PlantCARE is a database of plant *cis*-acting regulatory elements, enhancers and repressors, and is widely used to study the mechanisms of plant gene expression regulation (https://bioinformatics.psb.ugent.be/webtools/plantcare/html/). After the submission sequence runs, the user will receive an email containing the result file. From this perspective, it is quite convenient. However, the file size limit for uploading sequences is 100kb. In addition, the information in the result file cannot be directly used to draw images and display them. Firstly, the result of each promoter subsequence is a compressed file, and secondly, the information contained in the result file is relatively large. These data need to be filtered and integrated before they can be used for image display. These are very difficult for ordinary researchers. BioVizSeq can automatically split and upload sequence files larger than 100kb, filter the resulting files directly merged by users, and then draw publication level figures.

#### 2.4.8 Advance plot

**Figure 5:**
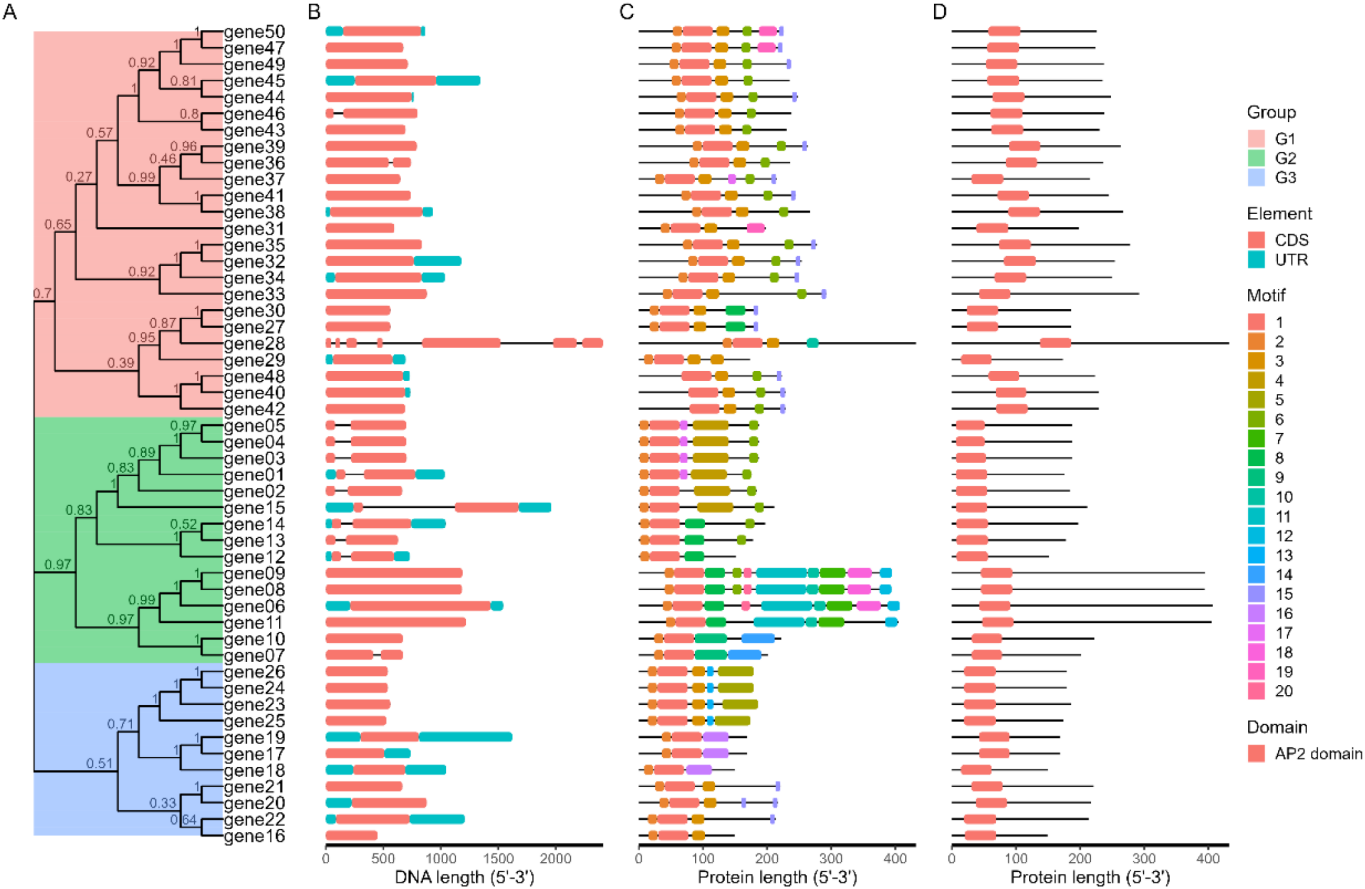
The Advance Plot function. Researchers can combine multiple types of results together when analyzing gene families. **(A)** The phylogenetic tree. **(B)** GFF/GTF structure. **(C)** MEME result. **(D)** PFAM result.

Researchers often combine multiple types of images together when analyzing gene families, such as evolutionary tree + gene structure + MEME motif + SMART domain. Therefore, we have specifically developed a function: combi_p. The result of combi_p is a list containing multiple graphic files, and users can freely combine the graphics within it. At the same time, based on this function and patchwork package (https://cran.r-project.org/package=patchwork), we have written a module in shinyapp specifically designed to facilitate users in combining graphics (**Figure 5**).

#### 2.4.9 Others

**Figure 6:**
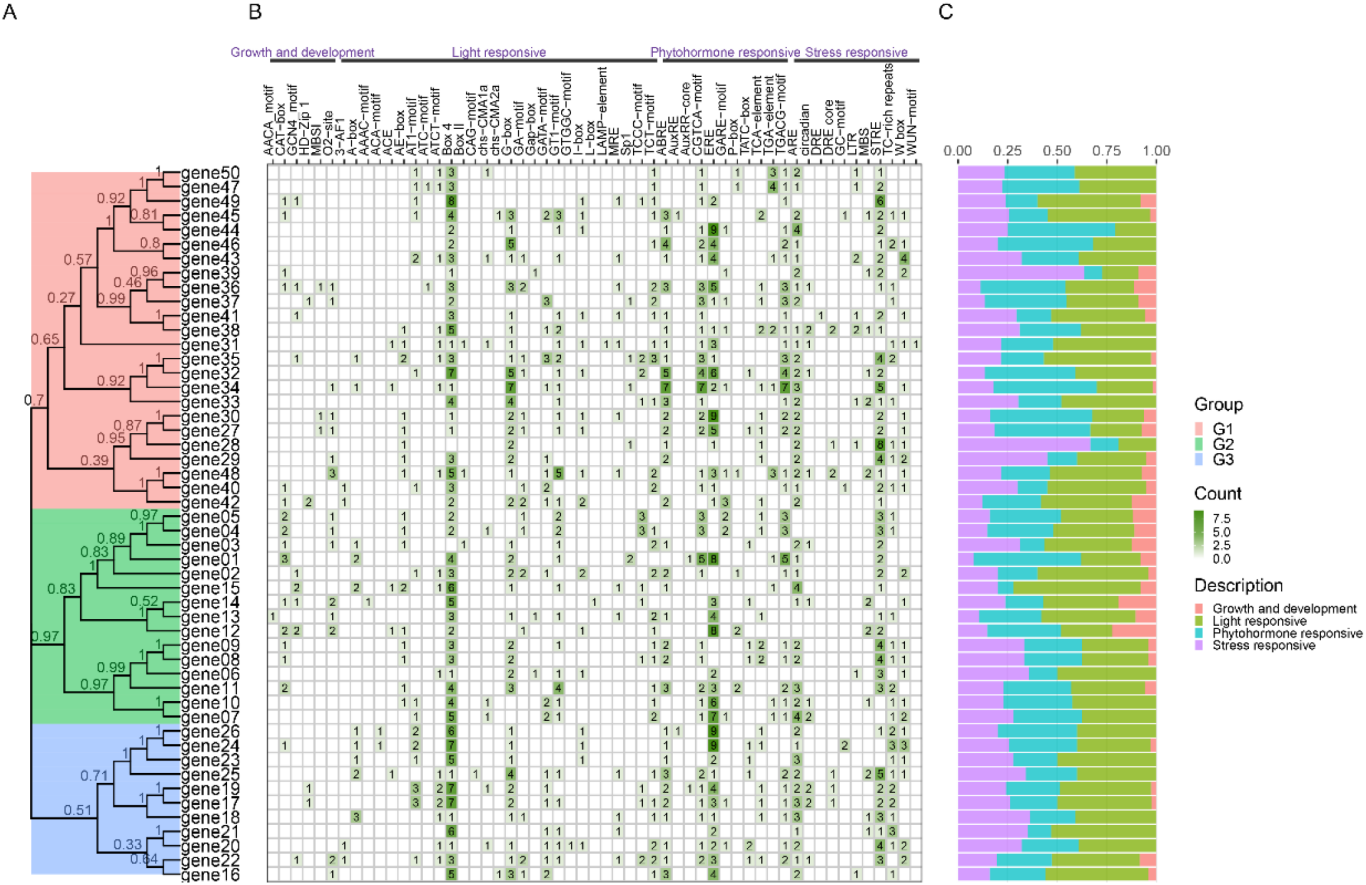
Combination diagram of types and numbers of *cis* regulatory elements on gene promoters. **(A)** The phylogenetic tree. **(B)** The diferent intensity colors and numbers of the grid indicate the numbers of different promoter elements in genes. **(C)** The different colored histogram represents the sum or percent of the *cis*-acting elements in each category.

In addition, we have also developed some other features, such as Advance Plantcare, to display the types and quantity distribution of cis acting elements on the promoter (**Figure 6**). The reason for developing this feature is that there are many types and quantities of cis acting elements on the promoter, and if we use Basic plot, it is easy to overlap and difficult to observe. For genes, we often conduct statistical analysis on their coordinates, sequence length, number of exons/introns, and analyze a series of physicochemical properties of the encoded protein sequence. Therefore, we have also added these two functions in Others, where users can analyze and download the results.

## 3 Conclusion

The BioVizSeq package provides a range of functions for analyzing and displaying components on biological sequences, such as gene structure, motif, structural domains and *cis* regulatory elements. At the same time, a local shinyApp with a user-friendly interface and online analysis services are provided, which greatly facilitates researchers without the need for coding capabilities.

## 4 Methods

The BioVizSeq package is developed using the R v4.3.1 (Team R. C, 2025) and utilizes the roxygen2 v7.2.3 package for generating and updating API documentation. The correctness of functions and the integrity of the package are verified using the devtools v2.4.5 package. The compilation is performed based on the DESCRIPTION and NAMESPACE files. The API documentation and website are built using pkgdown v2.0.7 with the _pkgdown.yml configuration file, which provides online help documentation (https://zhaosq2022.github.io/BioVizSeq/). For data manipulation, the seqinr v4.2–36 and stringr v1.5.0 are used to process fasta files and strings, respectively. The dplyr v1.1.4, and tidyr v1.3.0 packages from the tidyverse ecosystem are used to transform data structures. BioVizSeq prioritizes the use of ggplot2 v3.5.0, a widely used package that offers customizable visualization capabilities. In the end, we developed a series of functions for data analysis and graphical presentation (**Table 2**).

**Table 2:** Major functions of BioVizSeq.

Function	Description
Biovizseq	BioVizSeq shiny app start function
geom_rrect	A geom layer that can generate rounded rectangles
motif_plot	Basic plot to visualize the elements within bio-sequences
gff_to_loc	Extract the information of element from gff/gtf file
gff_plot	Visualization of element in gff/gtf file
meme_to_loc	Extract the information of elements from meme/mast file
meme_plot	Visualization of element in meme/mast file
meme_seq	Extract the motif sequence from meme/mast file
pfam_to_loc	Extract the information of domain from Pfam file
pfam_plot	Visualization of element in Pfam file
cdd_to_loc	Extract the information of domain from CDD file
cdd_plot	Visualization of element in CDD file
smart_to_loc	Extract the information of domain from SMART file
smart_plot	Visualization of element in SMART file
upload_fa_to_plantcare	Upload the fasta file to Plantcare and return the result
plantcare_classify	Classify the elements in the plantcare results
plantcare_to_loc	Extract the information of element from Plantcare file
plantcare_plot	Visualization of element in Plantcare file
combi_p	Get ggplot2 files to facilitate free combination

## Data Availability

The original contributions presented in the study are included in the article/supplementary material. Further inquiries can be directed to the corresponding authors.
